# Interference in thyroid function tests using the electrochemiluminescence immunoassay

**DOI:** 10.1530/ETJ-25-0396

**Published:** 2026-03-26

**Authors:** Chisako Imamura, Eijun Nishihara, Shuji Fukata, Mitsuru Ito, Mitsushige Nishikawa, Akira Miyauchi, Takashi Akamizu

**Affiliations:** Center for Excellence in Thyroid Care, Kuma Hospital, Kobe, Japan

**Keywords:** assay interference, thyroid function test, electrochemiluminescence immunoassay, anti-T3 monoclonal antibodies, macro-thyroid-stimulating hormone

## Abstract

**Objective:**

Electrochemiluminescence immunoassays (ECLIAs) are widely used for thyroid function testing but may be affected by rare assay interferences that can lead to misdiagnosis. We aimed to investigate the frequency and types of assay interferences in thyrotropin (TSH), free thyroxine (FT4), and free triiodothyronine (FT3) measurements using the Roche ECLIA.

**Methods:**

Between 2019 and 2023, 124,615 patients underwent thyroid function testing. Assay interference was suspected in 283 cases based on clinical–laboratory mismatch, thyroid hormone imbalance, or discrepancies between assay methods. Further validation assessed potential interferences.

**Results:**

Of the total tests (TSH: 124,609; FT4: 116,012; and FT3: 83,044), 71 interference cases were confirmed (TSH: 11 (0.009%), FT4: 6 (0.005%), and FT3: 53 (0.064%)). FT3 interferences were mainly due to idiotype and anti-streptavidin antibodies. Macro-TSH was detected in six cases and often led to unnecessary levothyroxine treatment. Furthermore, most interferences were detected by recognizing hormonal imbalances rather than clinical symptoms.

**Conclusion:**

FT3 tests are most prone to assay interference. Awareness of inter ference patterns and close collaboration between physicians and laboratory staff are essential to prevent diagnostic errors.

## Introduction

Measuring circulating free thyroid hormones and thyrotropin (TSH) is essential for evaluating thyroid function. Currently, immunoassay platforms are the most commonly used methods for thyroid function testing in clinical laboratories, particularly due to their fully automated operation, short turnaround time, and high specificity and sensitivity for different analytes. However, these assays are susceptible to interference. These interferences are minimized by advanced assay design but might still occur in rare cases. The frequency of interferences varies by hormone and measurement method, with reported rates approaching 1% (1, 2, 3). Approximately half of patients diagnosed with interferences undergo unnecessary additional testing and treatment (1).

Electrochemiluminescence immunoassay (ECLIA), a fully automated high-sensitivity immunoassay, uses various reagents, such as streptavidin-coated magnetic particles, biotin, and ruthenium-labeled antibodies. Autoantibodies against these molecules and excessive biotin intake can cause assay interference in the Roche ECLIA (1). Manufacturers have attempted to minimize interference by adding absorbing reagents (such as increasing tolerance to higher biotin intake) or improving labeling substances; however, interferences may still occur despite these modifications.

The frequency of interference in free thyroid hormone and TSH measurements using this specific ECLIA (Roche assay), as well as the clinical features of affected cases, has not been adequately verified in large cohorts. Therefore, in this study, we aimed to establish validation procedures for identifying assay interference in routine clinical practice and to characterize the clinical features of cases with various types of interference.

## Materials and methods

### Subjects

**Figure 1:**
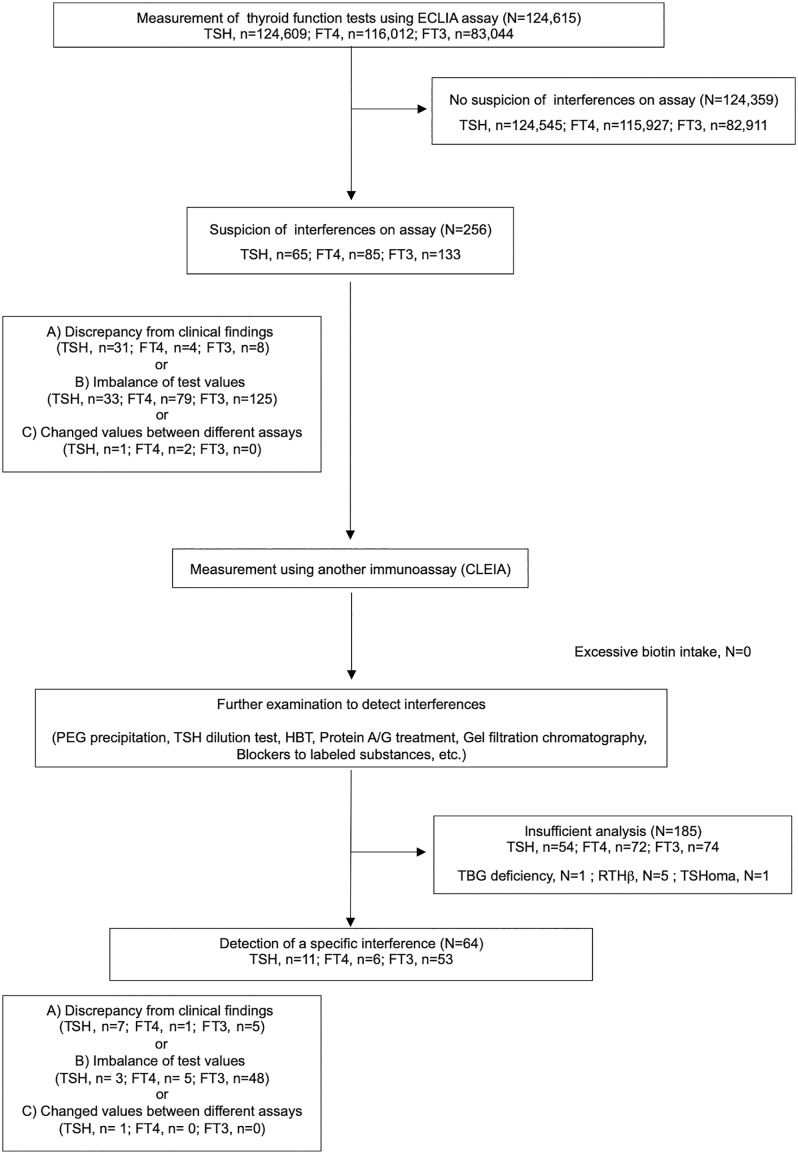
Flow chart of detection of assay interferences in thyroid function tests. All numbers, with or without assay interference, are presented as number of individual patients (*n*) and tested samples (*n*). When a participant was measured multiple times, the maximal discordant result was treated as a single case.

Between May 2019 and April 2023, 124,615 patients who visited Kuma Hospital for evaluation of various thyroid diseases and underwent thyroid function testing using the Roche ECLIA were consecutively enrolled. All counts, with or without assay interference, are presented as number of individual patients (*n*) and tested samples (*n*) in Fig. 1. When a participant underwent multiple measurements, the maximal discordant result was treated as a single case. Dietary supplement use or biotin intake was confirmed during medical interviews. The Ethics Committee of Kuma Hospital approved this study. Because the research involved the use of existing clinical data and blood samples stored at −80°C without freeze–thaw cycles before re-assay, the need for individual informed consent was waived, and an opt-out consent process was implemented (institutional review board approval number: 20250612-3).

### Thyroid function tests

Routine measurements of TSH, free thyroxine (FT4), and free triiodothyronine (FT3) were performed using the Elecsys ECLIA (Roche Diagnostics GmbH, Switzerland). The Accuraseed chemiluminescent enzyme immunoassay (CLEIA, FUJIFILM Wako Pure Chemical Corp, Japan) was used to re-evaluate thyroid function tests at Kuma Hospital (Fig. 1). Anti-thyroid peroxidase antibodies (TPOAbs) and anti-thyroglobulin antibodies (TgAbs) were measured using the Elecsys assay (Roche Diagnostics GmbH). The Roche ECLIA is a one-step assay, whereas the FUJIFILM Wako CLEIA is a two-step assay that is less susceptible to many interfering substances.

### Procedure for selecting possible participants with assay interferences

Possible cases of assay interference were extracted using the three criteria shown in Fig. 1: A) a discrepancy between thyroid hormone levels and clinical findings (e.g., an asymptomatic patient with laboratory values indicating significant thyrotoxicosis), B) an imbalance between thyroid hormone and TSH results, defined based on the distribution of previously identified interference samples (Supplementary Table 1 (see section on Supplementary materials given at the end of the article)), and C) significantly different thyroid hormone or TSH values obtained using different assay methods (i.e., one value falls within the reference interval, while the other is elevated, or a discrepancy of ≥1.5-fold between the two measurements) at another hospital or previously at our hospital. Possible interference was verified through a series of examinations, as described below.

### Polyethylene glycol precipitation

Equal volumes (300 μL) of 25% polyethylene glycol (PEG)-6000 (FUJIFILM Wako Pure Chemicals) were added to patient and control samples. Subsequently, the samples were incubated for 30 min at 20–25°C, centrifuged at 12,100 ***g*** for 10 min, and the supernatant was measured to calculate the recovery rate. Recovery (%) was calculated as follows: (measured value after PEG treatment × 2/measured value before PEG treatment) × 100. All samples with suspected interference in thyroid hormone and TSH assays were examined by PEG precipitation at Kuma Hospital. The reference ranges (99% confidence intervals (CIs)) were as follows – TSH: 20–65%, FT4: 140–180%, and FT3: 120–180%.

### TSH dilution test

The sample was diluted two-, five-, and tenfold, and TSH was measured using the Roche ECLIA at Kuma Hospital. Recovery (%) was calculated as follows: (measured value of the diluted sample/measured value before dilution) × 100. The limit of quantitation ranged from 0.005 to 100 μIU/mL. The intra-assay coefficients of variation were ≤10% for the TSH assay (4). The measured values of each diluted sample, prior to regression analysis, were within ±20% of the theoretical values. If linearity is not observed upon dilution, the presence of interfering substances is likely.

### Agarose beads protein A/G precipitation

Protein A (Sigma, Germany, Code No. 11134515001) or protein G (Sigma, Code No. 11243233001) agarose beads were used. Protein A or G was added in the same manner as in the PEG test to evaluate potential interference due to immunoglobulin G. Equal volumes (200 μL) of protein A or G were added to patient and control samples. The samples were incubated for 3 h at 4°C and centrifuged at 12,100 ***g*** for 5 min, and the supernatant was measured to calculate the recovery rate. Recovery (%) was calculated as follows: (measured value after protein A/G treatment × 2/measured value before protein A/G treatment) × 100. Samples that deviated by ≥15% from the recovery rate of the concurrently measured control sample were considered abnormal.

### Heterophilic antibody blocking tube treatment

Heterophilic antibody blocking tubes (HBTs; Scantibodies Laboratory, Inc., USA) were used to assess immunoglobulin M-type interference. The heterophilic blocking tube (Scantibodies Laboratory, product code 3IX762) was tapped to settle the lyophilized powder to the bottom of the tube, after which 200 μL of each sample were added. Samples were mixed by inverting five times and incubated at room temperature for 1 h. After mixing by pipetting several times, the samples were measured to evaluate interference caused by heterophilic antibodies. Recovery (%) was calculated as follows: (measured value after HBT treatment/measured value before HBT treatment) × 100. Samples that deviated by ≥15% from the recovery rate of the concurrently measured control sample were considered abnormal.

### Gel filtration analysis

Shodex Protein KW-804 columns (Showa Denko, Japan) were used for evaluation under neutral (pH 7.2) and acidic (pH 3.0) conditions. First, 200 μL of patient sample were eluted under neutral conditions with 150 mM sodium chloride and 50 mM Tris buffer. Then, acidic gel filtration was performed to elute fractions with high molecular weight TSH peaks using 200 μL of the collected fraction. The flow rate was 0.75 mL/min, and the fractionation time was 35 min (5).

### Detection of specific antibodies

Anti-streptavidin, anti-T3 or anti-T4 idiotype, anti-adsorption reagent, anti–ruthenium sulfonate (RuS) complex, and anti-ruthenium antibodies were analyzed and reported by Roche Diagnostics staff who were blinded to the clinical information.

### Statistical analysis

Comparisons of sex, TgAb, TPOAb, and initial contribution prevalence were performed using Fisher’s exact test. Differences in age and FT3 levels among interference groups were evaluated using the Kruskal–Wallis and Steel–Dwass tests. Differences were considered statistically significant at *P* <0.05.

## Results

The total number of thyroid function tests performed was as follows: TSH: 124,609; FT4: 116,012; and FT3: 83,044 cases. Further evaluation for suspected assay interference (TSH: 65, FT4: 85, and FT3: 133) was conducted in cases meeting at least one of the predefined criteria (Materials and methods and Fig. 1). After a series of verification tests, 71 cases of assay interference were detected, including TSH: 11 (0.009%; 95% CI: 0.005–0.016), FT4: 6 (0.005%; 95% CI: 0.001–0.013), and FT3: 53 (0.064%; 95% CI: 0.050–0.080) (Fig. 1 and Table 1). Among the identified causes of interference, idiotype antibodies against anti-T3 monoclonal antibodies in the FT3 measurement reagent were most prevalent (*n* = 23), followed by anti-streptavidin antibodies in the FT3 measurement reagent (*n* = 12) and macro-TSH (*n* = 6). Twenty cases were attributed to heterophilic antibodies against unknown antigens, affecting FT3 (*n* = 13), FT4 (*n* = 3), and TSH (*n* = 4) assays.

**Table 1:** Frequency of interference in thyroid function tests using the Roche ECLIA. All numbers with or without assay interference are presented as tested samples (*n*). When a participant was measured multiple times, the maximal discordant result was treated as a single case.

	Free T3	Free T4	TSH
Total evaluated cases	83,044	116,012	124,609
Interferences on assay	53 (0.064%)	6 (0.005%)	11 (0.009%)
Classification of causes			
Anti-T3 Ab	23		
Heterophilic Ab	13	3	4
Anti-SA Ab	12		
Anti-RuS Ab	3	3	
Anti-AR Ab	2		
Anti-Ru Ab			1
Macro-TSH			6

Anti-T3 Ab, idiotype antibodies against anti-T3 monoclonal antibodies; anti-SA Ab, anti-streptavidin antibodies; anti-AR Ab, anti-adsorption reagent antibodies; anti-RuS Ab, anti-ruthenium sulfonate complex antibodies; anti-Ru Ab, anti-ruthenium antibodies, T3, triiodothyronine; T4, thyroxine; and TSH, thyroid-stimulating hormone.

Subsequently, we compared the clinical features of the three most frequently observed FT3 interferences in the Roche ECLIA (Table 2 and Supplementary Tables 2–4). The cases with anti-streptavidin antibodies were relatively younger, and those with idiotype antibodies against anti-T3 monoclonal antibodies showed mildly elevated FT3 values (highest value: 5.66 pg/mL). The prevalence of anti-thyroid antibodies (TPOAb and/or TgAb) in cases with idiotype antibodies was much lower than that in the other groups. The initial identification of suspected interference by physicians or laboratory remarks was statistically equal among the three interference types (Table 2).

**Table 2:** Comparison of the clinical features of the three interferences in FT3 measurement. Data are presented as *n* (%) or as median (IQR). Bold text denotes statistically significant differences between groups.

	TA	SA	HA	*P* value		
				TA vs SA	TA vs HA	SA vs HA
*n*	23	12	13			
Male:female	5 : 18	2 : 10	1 : 12	1.000	0.385	0.593
Age (years)	69 (56.0–79.0)	34 (28.8–62.5)	52 (38.0–63.0)	**0.017**	0.064	0.569
FT3 level (pg/mL)	4.41 (4.17–4.60)	5.39 (4.71–6.98)	5.24 (4.46–6.06)	**0.002**	0.056	0.875
TPOAb	6 (26.1)	11 (61.5)	7 (53.8)	**0.001**	0.052	0.593
TgAb	5 (21.7)	8 (61.5)	8 (61.5)	**0.004**	**0.038**	0.293
Interference suspected by physician	7 (30.4)	7 (58.3)	3 (23.1)	0.153	0.716	0.111

TA, idiotype for anti-T3 monoclonal antibodies; SA, anti-streptavidin antibodies; HA, heterophilic antibodies; FT3, free triiodothyronine; TPOAb, anti-thyroid peroxidase antibody; and TgAb, anti-thyroglobulin antibody.

Steel–Dwass test; age and FT3 level. Fisher’s exact test; sex, TPOAb, TgAb, and initial contribution for suspected inference.

All six cases with macro-TSH showed elevated TSH levels and were diagnosed with subclinical hypothyroidism (Table 3). Four of the six cases were already being treated with levothyroxine. After the diagnosis of macro-TSH, levothyroxine was discontinued in three of these four cases. Macro-TSH was identified not through the laboratory remarks but by physicians due to the lack of improvement in TSH levels despite levothyroxine administration (cases 3, 5, and 6) or imbalanced test results (cases 1, 2, and 4) (Table 3).

**Table 3:** Macro-TSH levels in TSH measurements.

Case	Age (years)	Sex	Initial examination		Examination on LT4 treatment			PEG recovery (%)	Interference suspected by	TgAb/TPOAb
			TSH (μIU/mL)	FT4 (ng/dL)	TSH (μIU/mL)	FT4 (ng/dL)	LT4 (μg/day)			
1	66	F	15.20	1.03	NT	NT	None	9	Physician	−/−
2	44	F	16.10	1.29	NT	NT	None	12	Physician	−/−
3	78	F	5.72	1.02	8.74	1.73	25	8	Physician	+/NT
4	65	F	NT	NT	8.85	1.46	50	17	Physician	+/+
5	51	M	26.50	1.13	20.00	1.14	75	8	Physician	−/−
6	62	M	15.37	1.11	6.25	1.72	100	4	Physician	−/−

M, male; F, female; NT, not tested; TgAb, anti-thyroglobulin antibody; TPOAb, anti-thyroid peroxidase antibody.

Reference intervals in the Roche ECLIA were as follows: TSH: 0.61–4.23 µIU/mL, FT3: 2.3–4.0 pg/mL, and FT4: 0.9–1.7 ng/dL; the reference interval for PEG recovery was 20–65%.

## Discussion

In this study, we investigated the frequency of interference in thyroid function tests (TSH, FT4, and FT3) in a large cohort of patients and elucidated their clinical presentations in routine practice at a single institution. Interference occurred more frequently in FT3 measurements (53 cases: 0.064%), despite FT3 having the lowest test volume. Idiotype antibodies against anti-T3 monoclonal antibodies in the FT3 measurement reagent and anti-streptavidin antibodies were particularly common, whereas no idiotype antibodies against anti-T4 or anti-streptavidin antibodies were detected among FT4 interferences. FT3 interferences had the highest frequency (53/133: 39.8%), and FT4 measurement had the lowest (6/85: 0.71%) (Fig. 1). Suspected and confirmed interference cases in FT3 were higher than those in FT4. This difference may reflect earlier improvements in FT4 assay reagents or the higher analytical sensitivity of the FT3 assay, which is designed to respond to even slight signal changes, to accurately measure extremely small amounts of FT3 present in blood. In the latter case, high sensitivity may amplify the impact of minute interferences, making FT3 measurements more susceptible to assay interference.

Thyroid function is generally evaluated by measuring TSH and FT4. However, in patients receiving levothyroxine after total thyroidectomy or with thyroid atrophy, FT3 measurement is more useful than FT4 for assessing euthyroid metabolism (4, 6). Therefore, for such patients, accurate FT3 values combined with TSH are critical for thyroid function assessment.

Assay interference has been reported with the Roche ECLIA (7, 8, 9, 10, 11, 12, 13, 14). Consequently, manufacturers have improved various reagents to reduce interference (15, 16). RuS were modified to reduce interference with ruthenium; however, antibodies against RuS were observed in FT3 and FT4 assays in this study and a previous report (17). Therefore, it may be difficult to develop labeled substances that completely prevent interference. In this study, heterophilic antibodies against ‘unknown antigens’ were identified as the cause of interference in FT3, FT4, and TSH assays in 20 samples. The specific antigens involved in these cases remain unidentified.

**Figure 2:**
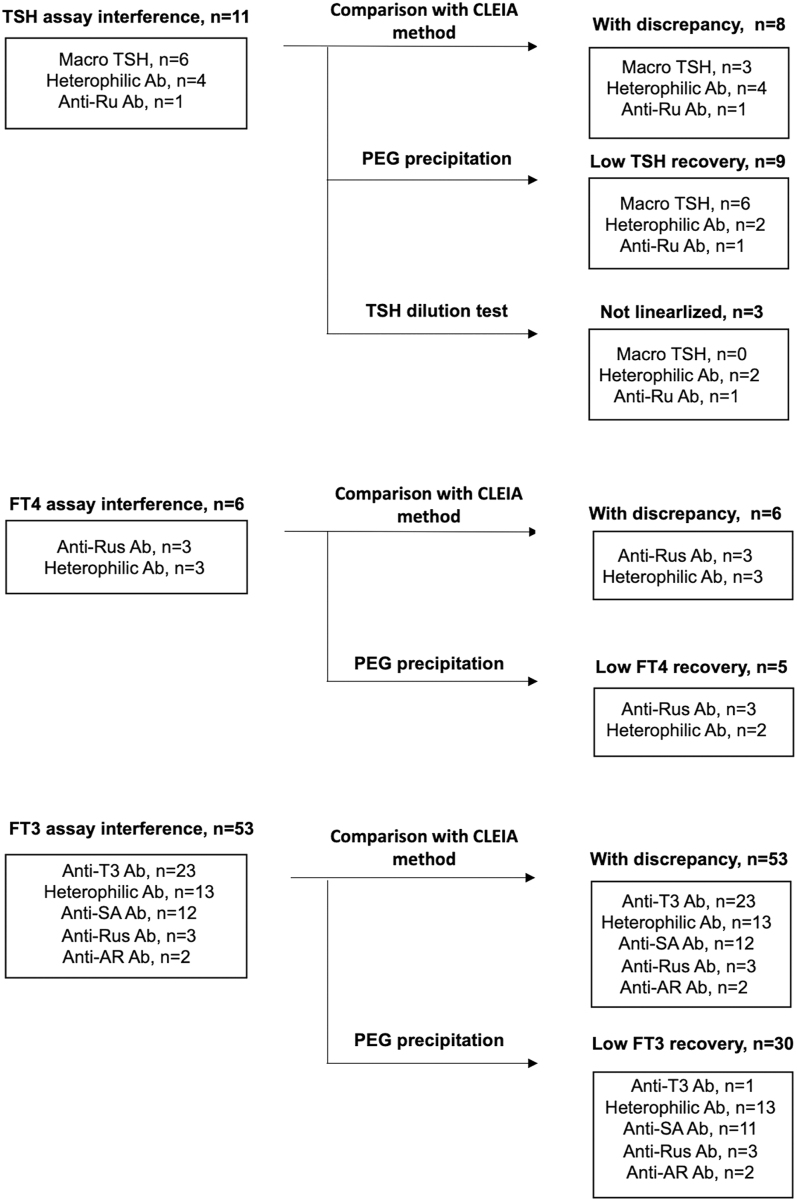
Ancillary tests to identify assay interferences among TSH and thyroid hormones.

Idiotype antibodies against anti-T3 monoclonal antibodies were more common in older patients and were associated with smaller increases in the FT3 level than those caused by anti-streptavidin antibodies, which may help physicians make a rough estimation before confirmatory testing (Table 2). In contrast, physician reports and laboratory remarks contributed equally to the initial identification of suspected interference (Table 2); however, some physicians did not request a detailed examination of potentially interfering samples until notified by the laboratory department. A key issue is how to determine assay interference more efficiently in the large volume of thyroid function tests. When there is any suspected interference based on the three criteria listed in the section titled Materials and methods (Fig. 1), almost all cases without macro-TSH exhibited discrepancies when measured using a different assay method (Fig. 2). To facilitate early identification of all potentially interfering samples with imbalanced values, our hospital recently implemented routine re-measurement using the FUJIFILM Wako CLEIA, a two-step immunoassay that is less susceptible to interference, before reporting results to physicians. In addition, PEG precipitation should be performed before detailed analyses, as it allows for cost-effective and easy confirmation of autoantibody binding. Combined verification using alternative assay measurements and PEG precipitation provides considerable assurance for the presence or absence of interference. For example, idiotype antibodies targeting anti-T3 monoclonal antibodies may be suggested by several findings, including discrepancies between two assays, normal FT3 recovery after PEG precipitation (Fig. 2), and clinical features (mildly elevated FT3 values and low frequency of anti-thyroid antibodies) (Table 2).

However, many cases of macro-TSH, which present as subclinical hypothyroidism, are already treated with levothyroxine replacement therapy (Table 3). Hence, laboratory staff may find it difficult to comment on potential assay interference. Macro-TSH was detected among 0.7–1.6% of cases with subclinical hypothyroidism (18, 19, 20). Physicians should recognize discrepancies between laboratory data and clinical findings, such as when TSH levels do not fully normalize despite an increased levothyroxine dose or when adverse clinical signs appear.

This study has some limitations. First, we selected cases for further examination based on clinical criteria, such as symptom–laboratory discrepancies or imbalanced measurements. Therefore, subtle interferences that did not meet these criteria may have been overlooked. Second, not all suspected assay interference cases underwent further examination (Fig. 1); thus, the frequency of each interference may be lower than previously reported (1, 2, 3). Among 185 patients categorized as having ‘insufficient analysis’, 69 showed discrepancies between ECLIA and CLEIA methods; however, insufficient sample volume precluded further testing. This number (*n* = 69) is similar to the number of patients in whom a specific interference was identified (*n* = 64), suggesting that the true number of interference cases may be approximately twice as high. Third, the definitive method for FT4 and FT3 measurement is equilibrium dialysis combined with liquid chromatography–tandem mass spectrometry; however, it could not be performed due to insufficient sample volume. Fourth, since this was a single-center study in which most patients had thyroid disease, and many were receiving anti-thyroid drugs or levothyroxine, interference may have been reported based on modified test data.

Assay interference in thyroid function testing was most frequently detected in FT3 assays and was most often identified through imbalances between TSH and thyroid hormone levels. Laboratory staff and physicians should be mindful of the possibility of abnormal results due to assay interference, to prevent misdiagnosis.

## Supplementary materials



## Declaration of interest

The authors declare that there is no conflict of interest that could be perceived as prejudicing the impartiality of the work reported.

## Funding

This research did not receive any specific grant from any funding agency in the public, commercial, or not-for-profit sector.

## Author contribution statement

CI and EN conceived the study and wrote the manuscript. CI and EN contributed to the acquisition and analysis of data. SF, MI, MN, AM, and TA critically reviewed the manuscript. All authors discussed the results and approved the final version of the manuscript.

## Data statement

Original data generated and analyzed during this study are included in this published article.
