# Supplementary material for: Interference in thyroid function tests using the electrochemiluminescence immunoassay
Source: Eur Thyroid J. 2026 Mar 26;15(2):ETJ250396. doi: 10.1530/ETJ-25-0396 (PMC13052764; doi:10.1530/ETJ-25-0396)
Supplement: Supplementary file 1 [file supplementary_materials.pdf]

Supplementary Table 1. Criteria of imbalanced thyroid tests in Kuma Hospital

	Measurement using the Roche ECLIA
1	0.1≦TSH < 0.5 and FT4 < 0.9
2	0.1≦TSH < 0.5 and FT4 > 2.2
3	0.5≦TSH < 3.0 and FT4 < 0.8
4	0.5≦TSH < 3.0 and FT4 > 1.9
5	3.0≦TSH < 10.0 and FT4 < 0.7
6	3.0≦TSH < 10.0 and FT4 > 2.0
7	0.1≦TSH < 0.5 and FT3 < 1.0
8	0.5≦TSH < 10.0 and FT3 < 1.9
9	0.5≦TSH < 3.0 and FT3 > 4.5
10	3.0≦TSH < 10.0 and FT3 > 4.0

ECLIA, Electrochemiluminescence Immunoassay

Reference intervals for TSH, FT3 and FT4 in the Roche ECLIA was 0.61-4.23 μIU/mL, 2.3-4.0 pg/mL and 0.9-1.7 ng/dL.

Supplementary Table 2. Classification of interference causes and respective examined methods

Classification of causes	Combination of examined methods
Anti-T3 Ab	Changed assay (CLEIA two-step assay), PEG precipitation, HBT, agarose beads protein A/G precipitation, changed reagent of FT3 detection antibodies (from monoclonal to polyclonal antibodies)
Anti-SA Ab	Changed assay (CLEIA two-step assay), PEG precipitation, HBT, agarose beads protein A/G precipitation, Pre-adsorption with streptavidin-coated magnetic particles
Anti-AR Ab	Changed assay (CLEIA two-step assay), PEG precipitation, HBT, agarose beads protein A/G precipitation, Gel filtration analysis, additive of interference eliminating protein
Anti-RuS Ab	Changed assay (CLEIA two-step assay), PEG precipitation, HBT, agarose beads protein A/G precipitation, Gel filtration analysis, changed reagent from sulfonated ruthenium complex labeling to ruthenium complex labeling
Anti-Ru Ab	Changed assay (CLEIA two-step assay), PEG precipitation, HBT, agarose beads protein A/G precipitation, Gel filtration analysis, changed reagent from ruthenium complex labeling to sulfonated ruthenium complex labeling
Macro-TSH	Changed assay (CLEIA two-step assay), PEG precipitation, HBT, agarose beads protein A/G precipitation, Gel filtration analysis (neutral and acidic conditions)
Heterophilic Ab	Changed assay (CLEIA two-step assay), PEG precipitation, HBT, agarose beads protein A/G precipitation, Gel filtration analysis

Anti-T3 Ab, Idiotype antibodies against anti-T3 monoclonal antibodies; Anti-SA Ab, Anti-streptavidin antibodies; Anti-AR Ab, Anti-adsorption reagent antibodies; Anti-Rus Ab, Anti-Ruthenium sulfonate complex antibodies; Anti-Ru, Anti-ruthenium antibodies;
CLEIA, chemiluminescent enzyme immunoassay; PEG, Polyethylene glycol; HBT, Heterophilic antibody blocking tubes

In these combined analyses, we defined "heterophilic antibodies for unknown antigens" as cases in which the HBT test was positive and no other interference was identified.

Supplementary Table 3. Idiotype antibodies against anti-T3 monoclonal antibodies in FT3 measurement

Case	Age	Sex	Roche ECLIA			FUJIFILM Wako CLEIA			Treatment	Initial identification	TPOAb/TgAb
			TSH (μIU/mL)	FT3 (pg/mL)	FT4 (ng/dL)	TSH (μIU/mL)	FT3 (pg/mL)	FT4 (ng/dL)			
1	81	M	23.20	5.66	1.10	20.10	1.01	1.12	None	Physician	＋/＋
2	43	M	2.48	4.83	1.30	3.01	1.37	1.31	None	Laboratory remarks	NT/NT
3	59	M	4.50	4.49	NT	4.20	1.61	NT	None	Laboratory remarks	－/－
4	54	M	3.16	4.41	1.18	3.14	3.28	1.19	None	Laboratory remarks	－/＋
5	80	F	7.12	4.29	NT	5.50	0.96	NT	None	Laboratory remarks	－/－
6	31	F	6.13	4.17	NT	4.89	0.94	NT	None	Laboratory remarks	－/－
7	85	M	1.41	4.58	1.23	1.17	1.52	1.27	LT4 37.5μg/day	Laboratory remarks	－/＋
8	78	M	1.06	4.50	1.86	0.94	0.93	1.92	LT4 75μg/day	Physician	NT/NT
9	87	M	1.10	4.44	1.52	0.96	1.30	1.43	LT4 75μg/day	Laboratory remarks	－/－
10	73	M	6.12	4.40	1.38	5.98	0.64	1.29	LT4 75μg/day	Laboratory remarks	－/－
11	69	M	0.55	4.16	NT	NT	1.46	NT	LT4 87.5μg/day	Physician	－/－
12	71	M	8.30	4.15	NT	7.76	1.18	NT	LT4 87.5μg/day	Laboratory remarks	－/－
13	73	F	0.65	4.76	2.07	0.60	1.46	2.09	LT4 87.5μg/day	Physician	－/－
14	58	M	0.78	4.44	1.57	0.78	1.75	1.47	LT4 100μg/day	Physician	－/－
15	60	F	1.44	4.90	1.75	1.35	1.44	1.73	LT4 100μg/day	Laboratory remarks	－/－
16	82	M	3.87	4.17	1.83	3.32	1.56	1.81	LT4 100μg/day	Laboratory remarks	－/－
17	72	M	1.80	4.11	NT	NT	NT	NT	LT4 100μg/day	Physician	－/－
18	68	M	1.31	3.37	1.74	1.30	1.86	1.63	LT4 125μg/day	Physician	－/－
19	64	M	4.64	4.26	1.19	4.68	1.80	1.17	MMI 1.25mg/day	Laboratory remarks	＋/＋
20	54	M	3.74	4.62	1.31	3.81	0.87	1.19	MMI 2.5mg/day	Laboratory remarks	＋/－
21	83	M	3.88	4.19	1.04	3.77	2.10	1.03	MMI 2.5mg/day	Laboratory remarks	＋/＋
22	32	M	5.02	4.09	1.16	5.39	1.16	NT	MMI 2.5mg/day	Laboratory remarks	＋/－
23	51	M	3.46	4.68	1.24	3.75	1.53	1.26	MMI 10mg/day	Laboratory remarks	＋/－

NT, not tested; LT4, levothyroxine; MMI, thiamazole; TPOAb, Anti-thyroid peroxidase antibody; TgAb, Anti-thyroglobulin antibody

Reference intervals in Roche ECLIA for TSH, FT3 and FT4 are 0.61-4.23 μIU/mL, 2.3-4.0 pg/mL and 0.9-1.7 ng/dL, respectively.

Reference intervals in FUJIFILM Wako CLEIA for TSH, FT3 and FT4 are 0.61-4.23 μIU/mL, 2.51-4.16 pg/mL and 0.83-1.77 ng/dL, respectively.

Supplementary Table 4. Anti-streptavidin antibodies in FT3 measurement

Case	Age	Sex	Roche ECLIA			FUJIFILM Wako CLEIA			Treatment	Initial identification	TPOAb/TgAb
			TSH (μIU/mL)	FT3 (pg/mL)	FT4 (ng/dL)	TSH (μIU/mL)	FT3 (pg/mL)	FT4 (ng/dL)			
1	25	F	1.07	6.90	1.39	1.06	3.45	1.72	None	Physician	＋/＋
2	31	F	6.91	13.50	0.88	8.61	3.09	NT	None	Laboratory remarks	＋/＋
3	35	F	0.87	7.22	1.44	1.13	2.81	1.35	None	Laboratory remarks	＋/＋
4	30	F	3.24	4.71	1.79	2.96	4.24	1.53	None	Physician	＋/＋
5	45	F	0.43	4.78	1.71	0.53	2.99	1.34	None	Physician	＋/＋
6	64	M	0.85	4.69	1.82	0.85	2.90	1.78	None	Physician	＋/＋
7	62	F	0.12	4.37	NT	0.14	2.51	1.74	LT4 75μg/day	Physician	−/−
8	84	F	＞100.0	4.36	0.51	＞100.0	1.40	0.65	LT4 100μg/day	Physician	＋/＋
9	71	F	2.68	5.59	NT	2.14	3.16	NT	LT4 100μg/day	Laboratory remarks	＋/−
10	33	F	<0.005	5.99	2.50	0.01	3.23	2.13	LT4 137.5μg/day	Physician	＋/＋
11	23	M	3.47	8.22	1.24	5.72	3.16	NT	MMI 2.5mg/day	Laboratory remarks	＋/−
12	18	F	0.65	5.19	1.47	0.66	3.37	NT	MMI 2.5mg/day	Laboratory remarks	＋/−

NT, not tested; LT4, levothyroxine; MMI, thiamazole; TPOAb, Anti-thyroid peroxidase antibody; TgAb, Anti-thyroglobulin antibody

Reference intervals in Roche ECLIA for TSH, FT3 and FT4 are 0.61-4.23 μIU/mL, 2.3-4.0 pg/mL and 0.9-1.7 ng/dL, respectively.

Reference intervals in FUJIFILM Wako CLEIA for TSH, FT3 and FT4 are 0.61-4.23 μIU/mL, 2.51-4.16 pg/mL and 0.83-1.77 ng/dL, respectively.

Supplementary Table 5. Anti-heterophilic antibodies in FT3 measurement

Case	Age	Sex	Roche ECLIA			FUJIFILM Wako CLEIA			Treatment	Identification	TPOAb/TgAb
			TSH (µIU/mL)	FT3 (pg/mL)	FT4 (ng/dL)	TSH (µIU/mL)	FT3 (pg/mL)	FT4 (ng/dL)			
1	38	F	0.67	11.80	1.52	1.03	3.00	1.73	None	Laboratory remarks	＋/＋
2	52	F	0.81	4.46	1.18	0.93	2.84	1.25	None	Physician	＋/－
3	84	F	0.63	5.39	2.46	1.36	3.00	1.30	None	Laboratory remarks	＋/＋
4	41	F	1.37	4.06	2.10	1.41	3.24	1.29	None	Laboratory remarks	－/－
5	32	F	3.77	5.24	1.44	3.71	3.02	1.49	None	Physician	－/＋
6	51	F	0.69	5.00	1.23	0.68	2.83	NT	None	Laboratory remarks	＋/－
7	57	F	6.17	4.17	1.37	6.10	2.59	NT	LT4 62.5µg/day	Laboratory remarks	NT/－
8	58	F	1.92	4.51	1.48	1.53	2.24	1.76	LT4 75µg/day	Laboratory remarks	＋/＋
9	71	F	8.38	4.00	NT	6.58	2.61	NT	LT4 75µg/day	Laboratory remarks	NT/－
10	15	F	<0.005	8.77	NT	0.01	4.04	2.33	LT4 100µg/day	Physician	NT/＋
11	63	M	7.88	6.06	NT	8.17	2.83	NT	LT4 100µg/day	Laboratory remarks	－/＋
12	30	F	1.22	5.78	1.11	1.11	2.42	1.31	LT4 100µg/day	Laboratory remarks	＋/＋
13	72	F	0.81	10.20	>7.77	12.61	2.47	1.01	MMI 15mg+KI 50mg/day	Laboratory remarks	＋/＋

NT, not tested; LT4, levothyroxine; MMI, thiamazole; KI, potassium iodine; TPOAb, Anti-thyroid peroxidase antibody; TgAb, Anti-thyroglobulin antibody

Reference intervals in Roche ECLIA for TSH, FT3 and FT4 are 0.61-4.23 µIU/mL, 2.3-4.0 pg/mL and 0.9-1.7 ng/dL, respectively.

Reference intervals in FUJIFILM Wako CLEIA for TSH, FT3 and FT4 are 0.61-4.23 µIU/mL, 2.51-4.16 pg/mL and 0.83-1.77 ng/dL, respectively.
